# Impact of routine coronary catheterization in low extremity artery disease undergoing percutaneous transluminal angioplasty: study protocol for a multi-center randomized controlled trial

**DOI:** 10.1186/s13063-016-1237-0

**Published:** 2016-02-29

**Authors:** I-Chih Chen, Cheng-Han Lee, Ting-Hsing Chao, Wei-Kung Tseng, Tsung-Hsien Lin, Wen-Jung Chung, Jen-Kwan Li, Hsuan-Li Huang, Ping-Yen Liu, Ting-Kuang Chao, Chuin-Yuan Chu, Chih-Chan Lin, Po-Chao Hsu, Wen-Huang Lee, Po-Tseng Lee, Yi-Heng Li, Shih-Ya Tseng, Liang-Miin Tsai, Juey-Jen Hwang

**Affiliations:** Department of Internal Medicine, Tainan Municipal Hospital, Tainan, Taiwan; Department of Internal Medicine, National Cheng Kung University College of Medicine and Hospital, Tainan, Taiwan; Division of Cardiology, Department of Internal Medicine, National Cheng Kung University College of Medicine and Hospital, No. 138, Sheng-Li Road, North District, Tainan, 704 Taiwan; Division of Cardiology, Department of Internal Medicine, E-Da University College of Medicine and Hospital, Kaohsiung, Taiwan; Division of Cardiology, Department of Internal Medicine, Kaohsiung Medical University and Hospital, Kaohsiung, Taiwan; Division of Cardiology, Department of Internal Medicine, Chang-Gung Memorial Hospital Kaohsiung, Kaohsiung, Taiwan; Division of Cardiology, Department of Internal Medicine, National Taiwan University Hospital, Taipei, Taiwan; Division of Cardiology, Department of Internal Medicine, Buddhist Tzu Chi General Hospital, Taipei Branch, Taipei, Taiwan; Department of Otolaryngology, Far Eastern Memorial Hospital, New Taipei City, Taiwan; Division of Public Health and Preventive Medicine, Department of Medicine, Faculty of Medicine and Dentistry, University of Alberta, Edmonton, AB Canada

## Abstract

**Background:**

The prevalence of significant obstructive coronary artery disease with complex lesions is high in patients who have low extremity artery disease (LEAD). However, intermediate- or long-term cardiovascular prognosis of LEAD patients undergoing percutaneous transluminal angioplasty (PTA) remains poor. Accordingly, prophylactic coronary revascularization may modify short- and long-term cardiovascular outcomes of LEAD patients receiving PTA. Because myocardial ischemic symptoms are often masked in LEAD and the accuracy of non-invasive stress tests is usually limited, a high-quality randomized controlled trial aimed at the investigation of the prognostic role of coronary evaluation strategies before PTA is warranted.

**Methods/Design:**

The proposed study is designed as a prospective, multi-center, open-label, superiority, randomized controlled trial. The study is conducted in high-volume centers for PTA and coronary revascularization in Taiwan. To meet the inclusion criteria, the patients must be at least 20 years old, have known LEAD, and have been admitted for elective PTA. We plan to enroll 450 participants who are randomly allocated to a routine group (routine coronary angiography without a previous non-invasive stress test before PTA) and a selective group (selective coronary angiography based on the results of non-invasive stress tests before PTA) with 1:1 ratio. Besides, we expect to enroll about 250 additional participants, who are not willing to be randomly assigned, in the registration group. The choice of revascularization procedure depends on the operator’s or cardiovascular team’s suggestion and the patient’s decision. Clinical follow-up will be performed 30 days after PTA and every 6 months until the end of the 1-year follow-up for the last randomly assigned participant. The primary endpoint is the composite major adverse cardiac event on long-term follow-up. Pre-specified secondary and other endpoints are also evaluated. Those assessing biomarkers and clinical endpoints are all blinded after assignment to interventions.

**Discussion:**

The results of the trial will, for the first time, support better decision-making for coronary evaluation before PTA in LEAD. If favorable, routine coronary angiography followed by revascularization will improve cardiovascular outcomes in LEAD patients undergoing PTA.

**Trial registration:**

ClinicalTrials.gov identifier: NCT02169258 (registered on 21 June 2014); registry name: Routine Coronary Catheterization in Low Extremity Artery Disease Undergoing Percutaneous Transluminal Angioplasty (PIROUETTE-PTA).

**Electronic supplementary material:**

The online version of this article (doi:10.1186/s13063-016-1237-0) contains supplementary material, which is available to authorized users.

## Background

Patients with low extremity artery disease (LEAD) have a high risk of death from cardiovascular cause [1]. The existence and severity of coronary atherosclerosis are known to be correlated to those of peripheral artery disease [2–5]. The prevalence of significant obstructive coronary artery disease (CAD) is high (up to 70 %) in patients who have LEAD [2, 6]. Besides, complex coronary artery lesions are more common in patients with LEAD than in those without [5]. Furthermore, patients with concomitant LEAD are prone to have a greater chance of coronary disease progression [7]. Since myocardial infarction is a major cause of peri-operative death in vascular surgery [6], substantial efforts, such as risk stratification and routine or selective coronary angiography/revascularization before vascular surgery [8–11], have been made in order to improve cardiovascular outcome during operation. Despite inconsistent results in previous clinical studies [12–14], a recently published prospective randomized controlled trial (RCT) with 208 enrolled cases showed that a strategy of routine coronary angiography positively impacts long-term outcome of patients with peripheral artery disease at medium-high risk for vascular operation [6]. Nevertheless, coronary angiography/revascularization is recommended only in some clinical situations before vascular surgery by clinical guidelines [15–17].

Percutaneous transluminal angioplasty (PTA) is usually the first choice of revascularization for the majority of patients with LEAD in the current era. However, intermediate- or long-term prognosis of LEAD patients undergoing PTA remains poor; the average rate of total mortality is 25 % and the average rate of cardiovascular death is 12 % [18–21]. CAD is an independent predictor of total mortality after PTA [18]. Furthermore, ischemia/reperfusion injury after PTA can cause systemic inflammatory response [22, 23], probably leading to peri-procedural cardiovascular mortality in the existence of significant CAD [24]. Interestingly, silent ischemia is common in patients with LEAD because of the high prevalence of diabetes mellitus and inactivity in such patients. A commonly used stress test, the treadmill exercise test, for screening the existence of CAD is usually unsuitable in patients with LEAD because intermittent claudication with resultant limited activity or in bedridden status is frequently present due to severe peripheral artery disease. Furthermore, the accuracy of the available stress tests, including the treadmill exercise test, dobutamine stress echocardiography (DSE), or dipyridamole thallium 201 myocardial perfusion scintigraphy (dTS), is not so high (around 70–75 %), probably hampering vulnerable cases from receiving beneficial revascularization therapy, especially in asymptomatic CAD. Accordingly, prophylactic coronary revascularization may modify short- and long-term cardiovascular outcomes of LEAD patients receiving PTA. However, there is no RCT aimed at the investigation of the prognostic role of routine angiography and revascularization before PTA for LEAD. In addition, it has never been mentioned in the clinical guidelines. Therefore, there is substantial variability in practice strategies among interventionists. Taken together, further investigation of the clinical role of routine screening for the presence of CAD with subsequent revascularization has strong rationality and is deemed mandatory in LEAD patients undergoing PTA.

In this principal investigator-initiated prospective RCT, the PIROUETTE-PTA (Prognostic Impact of Routine Coronary Catheterization in Low Extremity Artery Disease Undergoing Percutaneous Transluminal Angioplasty) Study investigators will evaluate the prognostic effects of routine coronary angiography (routine strategy) versus selective coronary angiography based on the results of non-invasive stress tests (selective strategy) or conservative management (real-world strategy) before PTA for LEAD. The potential mechanisms will be elucidated. We hypothesize that a strategy of routine coronary angiography will positively impact long-term outcome as well as favorable cost-effectiveness, being superior to “selective strategy” or “real-world” strategy, in LEAD patients receiving PTA.

## Methods/Design

### Study design

The proposed study is designed as a prospective, multi-center, open-label, superiority RCT. This study protocol with amendment (version 5.0; 25 May 2014) has been approved by the institutional review board (IRB) of National Cheng Kung University Hospital (identifier: B-BR-103-023) and has been registered in ClinicalTrials.gov (identifier: NCT02169258). Provisions of all items from the World Health Organization Trial Registration Data Set can be found in this protocol and at www.clinicaltrials.gov. This study conforms to CONSORT (Consolidated Standards of Reporting Trials) 2010 guidelines for RCTs [25] and CONSORT extension for trials assessing non-pharmacologic intervention [26] (Additional file 1). The study protocol also fulfills SPIRIT (Standard Protocol Items: Recommendations for Interventional Trials) 2013 guidelines [27]; Additional file 2 shows this in more detail. All study participants give informed consent and this study follows the regulation of the ethics committee of the National Cheng Kung University Hospital. Any modifications to the protocol will require a formal amendment approved by the IRB of National Cheng Kung University Hospital and other participating sites. The investigators and study nurses will fully explain the study protocol and get the informed consent back from potential trial participants or authorized surrogates. An additional signed consent form must be obtained from all participants in sub-studies if information with respect to sample collection is not covered in the original informed consent form for the main PIROUETTE-PTA study. The study is conducted in high-volume centers (either academic medical centers or regional hospitals) for PTA and coronary revascularization in Taiwan. Reference to where list of study sites can be obtained at www.clinicaltrials.gov.

### Participants and eligibility

**Fig. 1:**
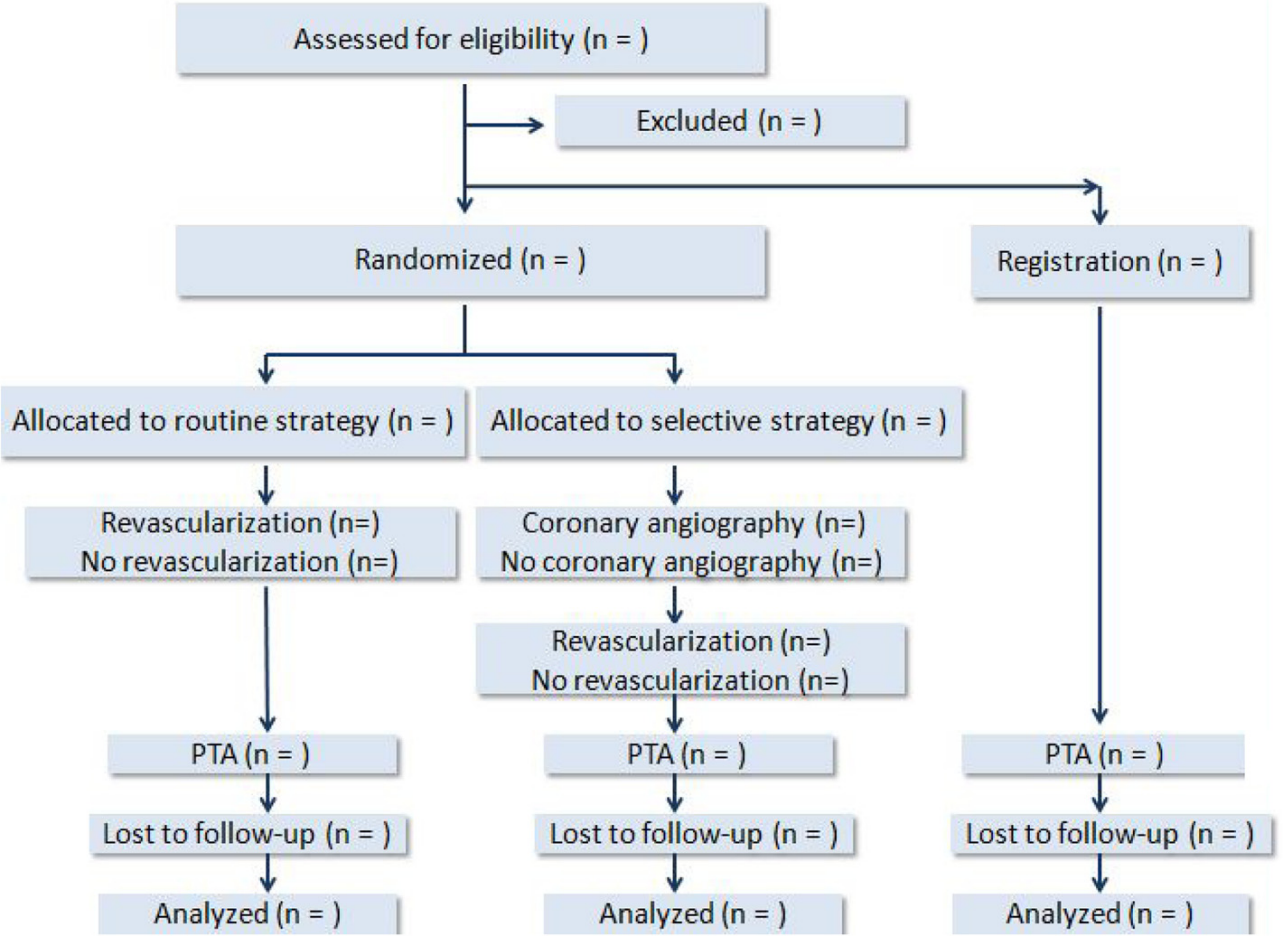
Participant flow diagram. *PTA* percutaneous transluminal angioplasy

This prospective RCT will consecutively enroll about 700 (450 randomly assigned and estimated 250 in registration strategy) eligible patients (≥20 years old) who have known LEAD (documented by previous angiography or sonography) and were admitted for elective PTA. The flow diagram is shown in Fig. 1. The definition of LEAD will be the presence of significant stenosis (≥70 % narrowing as compared with the reference vessel size), including total occlusion, in at least one major artery in the low extremities. Subjects are excluded if they have at least one of the following situations before screening: known CAD or unstable angina within the past 3 months, acute myocardial infarction within the past 6 months, known CAD having received percutaneous coronary intervention (PCI) or bypass surgery within the past 6 months, plan to do bypass surgery for known LEAD, pregnancy, documented active malignancy, and need for emergency PTA. The inclusion and exclusion criteria are listed in Table 1.

**Table 1:** Eligibility criteria

Inclusion criteria	Exclusion criteria
1. Must be at least 20 years old. Capable of giving written informed consent.	1. Known coronary artery disease or unstable angina within the past 3 months.
2. Agrees to comply with the study protocol.	2. Acute myocardial infarction within the past 6 months.
3. Known low extremity artery disease (documented by previous angiography or sonography) admitted for elective percutaneous transluminal angioplasty.	3. Known coronary artery disease having received percutaneous coronary intervention or bypass surgery within the past 6 months.
	4. Plans to do bypass surgery for known low extremity artery disease.
	5. Has been pregnant.
	6. Has documented active malignancy.
	7. Needs emergency percutaneous transluminal angioplasty.

### Eligibility criteria for investigating centers and those performing the interventions

The minimal requirements of the investigating centers are as follows: (1) a medical center or a regional hospital equipped with at least 500 acute care beds, (2) an experienced participating site where there is a good track record with respect to conducting clinical trials, and (3) a high-volume center with at least 500 PCI procedures and at least 100 PTAs each year. Those who are performing the interventions should fulfill both of the following eligibility criteria: (1) an experienced investigator who has a good track record with respect to conducting clinical trials and (2) an experienced operator with at least 75 PCI procedures and at least 50 PTAs each year.

### Blinding and randomization

Owing to the nature of the trial interventions, it will not be possible to blind the investigators at clinical trial sites, the participants, or the participants’ relatives. All other parties in the trial will be blinded. Those assessing biomarkers, single-nucleotide polymorphisms, micro-ribonucleic acids, and clinical endpoints are all blinded after assignment to interventions. Investigators conducting the statistical analyses will be blinded; the two intervention groups and one registry group will be coded as, for example, A, B, and C.

Eligible participants were randomly assigned to routine strategy or selective strategy by using unrestricted randomization and open label for allocation. Sealed envelopes for allocation concealment are provided. The allocation sequence is generated by Ting-Hsing Chao. Detailed information regarding the allocated group is given on cards contained in sequentially numbered, opaque, and sealed envelopes. These are prepared at the core coordinating center and kept in an appropriate location on each site. After enrollment of a participant, the appropriate numbered envelope is opened at the participating site. The information on whether the patient is to be a “routine” or “selective” case can be found on the card and this information then is given to the coordinator of the core coordinating center. Then, the investigator and research nurse follow the intervention protocol according to the allocation assignment. If participants are not willing to be randomly assigned, they will be included in the registration group if they agree. This information should also be given to the coordinator of the core coordinating center. The enrollment will be closed if the target number of randomly assigned participants is reached.

### Allocation and intervention

Participants allocated to the systemic strategy will receive routine coronary angiography before PTA without a previous non-invasive stress test, and subjects allocated to the selective strategy will undergo non-invasive evaluation of possible myocardial ischemia by using DSE or dTS followed by coronary angiography if the test is obviously positive for ischemia. Clinical decisions are reached by consensus of operators, patients, and family, as usual care in the registration group.

### Angiographic strategy

CAD is defined as significant luminal narrowing (≥50 % diameter stenosis as compared with the size of the adjacent reference vessel) in at least one of the coronary arteries (left main trunk, left anterior descending artery, left circumflex artery, and right coronary artery) or their major branches (diagonal branches, obtuse marginal branches, acute marginal branches, posterior descending artery, and posterior lateral branches). Based on the regulation of Taiwan National Health Insurance, significant CAD needing further revascularization is defined as luminal stenosis of at least 70 % of the major epicardial vessels and bypass graft vessel or their major branches (≥50 % for left main trunk or instent restenotic lesion) with reference vessel larger than 2.5 mm in diameter and moderate area of vulnerable myocardium for ischemia. Intermediate stenotic lesions, luminal stenosis of around 50 to 70 %, should be determined if they are hemodynamically significant by fractional flow reserve. Intermediate lesions with a fractional flow reserve of not more than 0.8 are considered hemodynamically significant and require further revascularization.

### Coronary revascularization strategy

The choice of revascularization procedure, either PCI or coronary artery bypass surgery, depends on the operator’s or cardiovascular team’s suggestion and the patient’s decision. A staged approach (myocardial revascularization followed by PTA) and simultaneous approach (PCI immediately followed by PTA at the same time if clinically suitable) are both allowed. However, PTA followed by myocardial revascularization within 21 days after PTA is also allowed in some participating sites because of local IRB request but should be limited to specific situations, in which the utmost benefit for patients is selected by clinical judgment. The duration from revascularization to PTA should be within 60 days. PCI is performed at the time of coronary angiography by using new-generation drug-eluting (more encouraging) or bare metal stents. Participants were put on dual anti-platelet treatment, including 100 mg aspirin and 75 mg clopidogrel per day, thereafter. Aspirin is used indefinitely, whereas add-on clopidogrel is used for at least 1 month in bare metal stent placement and 3 months in drug-eluting stent deployment. The usual practices of interventionists or surgeons with respect to evaluating the severity of CAD and selecting revascularization strategy are determined through literature review and through a survey of study interventionists or surgeons to achieve consensus. Each study interventionist or surgeon should agree to adhere to the aforementioned intervention protocol. All images of coronary angiography and PCI are recorded. A senior experienced interventionist who is not aware of assignments of study groups will review a sufficient number of the off-line images in an attempt to ensure the adherence of investigators with the protocol. There are no relevant concomitant care and interventions that should be prohibited during the trial.

### Blood sampling and measurement of biomarkers

Blood samples are obtained from peripheral arteries in all study subjects before PTA (20 ml) and after PTA (10 ml) and are prepared and stored for enzyme-linked immunosorbent assay as well as genotyping and measurement of micro-ribonucleic acids in plasma and mononuclear cells.

### Data collection and outcome measurement

The minimum follow-up period is 1 year and the maximum is 3 years. Clinical outcomes are obtained by chart review if the patient is still in the hospital and followed up by clinic visit at 30 days after indexed PTA if the patient has been discharged from the hospital and every 6 months thereafter. If participants miss indicated clinic visits, study assistants will call or directly contact the participants or subjects’ family.

The primary endpoint is the composite major adverse cardiac event (MACE) on long-term follow-up. Pre-specified secondary endpoints include the MACE between the screening and 30 days after PTA, and the composite major coronary event on long-term follow-up. Other endpoints include death from any cause, congestive heart failure requiring hospitalization, and any coronary revascularization. The MACE includes cardiovascular death, non-fatal myocardial infarction, non-fatal stroke, and target vessel revascularization (any unplanned coronary vessel revascularization). The composite major coronary event includes fatal or non-fatal myocardial infarction, recurrent angina pectoris, and target vessel revascularization (any unplanned coronary vessel revascularization). The diagnosis of myocardial infarction and stroke has been previously described in detail [6]. Clinical follow-up will be performed 30 days after PTA and every 6 months until the end of the 1-year follow-up for the last randomly assigned participant. The follow-up schedule is listed in Table 2. All outcome data should still be collected for participants who discontinue or deviate from intervention protocols. The clear definitions of each component of primary and pre-specified secondary endpoints are provided to all assessors, and training of assessors to enhance the quality of measurements is performed on site initiation visit.

**Table 2:** Follow-up schedule

	V0	Cath	PCI	CABG	PTA	V1	V2	V3	V4	V5	V6	V7	V8
Time elapse from PTA					0	30 (±7)D	24 (±2)W	48 (±2)W	72 (±2)W	96 (±2)W	120 (±2)W	144 (±2)W	168 (±2)W
CRF	001	002	003	004	005	006	007	008	009	010	011	012	013
Markers/SNPs/miRNAs					X								
CBC	X												
BCS	X												
BW/BH	X												
BP/HR	X					X	X	X	X	X	X	X	X
History and PE	X					X	X	X	X	X	X	X	X

*BCS* biochemistry study, *BH* body height, *BP* blood pressure, *BW* body weight, *CABG* coronary artery bypass grafting, *Cath* coronary catheterization, *CBC* complete blood cell count, *CRF* case report form, *D* days, *HR* heart rate, *miRNAs* micro-ribonucleic acids, *PCI* percutaneous coronary intervention, *PE* physical examination, *PTA* percutaneous transluminal angioplasty, *SNPs* single-nucleotide polymorphisms, *V* visit, *W* weeks

The pre-specified endpoints and serious adverse events (SAEs) should be reported to the data management committee and accompanied with required documents. The primary, secondary, and other endpoints are adjudicated on the basis of pre-specified criteria by an endpoint adjudication committee whose members are not aware of assignments of study groups. A conclusion is determined and achieved by voting or reaching consensus after discussion from members.

### Data management and monitoring

Electronic case report forms (CRFs) for the PIROUETTE-PTA trial are developed on the infrastructure of the Clinical Study Information System (CSIS) in cooperation between the coordinating investigator and a data manager at the Clinical Research Center of National Cheng Kung University Hospital. CSIS is the information management service provided by the Taiwan Clinical Trial Bioinformatics and Statistical Center, Training Center, and Pharmacogenomics Laboratory to facilitate the coordinating investigator in building up the electronic CRF system in investigator-initiated trials. This resource center is funded by the National Research Program for Biopharmaceuticals under grant support by the Ministry of Science and Technology (grant number 104-2325-B-002-033) and the Center for Systems and Synthetic Biology in National Yang-Ming University in Taiwan. Access to the CSIS will be possible around the clock every day and data can be entered continuously for all the randomly assigned patients. The coordinating investigator will have access to monitor the data input. If the entry is partially or completely missing or seems flawed on one or more randomly assigned patients, the coordinator will have the opportunity to contact the primary investigator in order to correct or complement data inputs to optimize the quality of the data. There are 13 CRFs to be filled on the CSIS at each indicated visit in the current study; Additional file 3 shows clinical data to be collected and recorded in more detail. The steering committee has access to the full trial dataset.

Original CRFs and source document will be kept on file and stored in numerical order and in a secure place with limited access at the participating site. All study-related records, materials, or documents are identified only by a coded identification number to keep participants’ confidentiality, whereas any records that should contain names or other personal identifiers, such as informed consent forms, will be stored separately from study records. An inspection team will select participating sites to monitor quality of the study at audit visits. Besides, a subset of the original CRFs will be requested for quality control during study. When a form is selected, the participating site staff will send the copy to the data management committee for inspection and double data entry.

We will provide a data and safety monitoring plan rather than establishing a data and safety monitoring committee because local IRBs consider that the interventions performed in the current study are medical routines. The data and safety monitoring plan includes reporting SAEs on time, increasing monitoring frequency, and analyzing safety data every 3 months. An interim analysis is performed on the primary endpoint by an independent statistician, who is blinded for the treatment allocation, when 50 % of patients have been randomly assigned and have completed 6-month follow-up. The statistician will report to the steering committee. The steering committee makes the final decision to terminate the trial and will report to the IRB of National Cheng Kung University Hospital. The trial will be ended by using symmetric stopping boundaries at a *P* value of less than 0.001. The trial will not be stopped in case of futility unless the statistician advises otherwise and the steering committee decides to terminate the trial for futility after discussion.

An adverse event that meets the criteria for an SAE between study enrollment and the end of the study will be reported to the local IRB. An SAE for this study is any untoward medical occurrence causing any of the following: a life-threatening condition, severe or permanent disability, or prolonged hospitalization. An SAE occurring after a participant withdraws consent from the study will not be reported unless the investigators consider that the event might have been caused by the protocol procedure. We will continue health care for trial participants regardless of having potential trial-related harm.

### Strategies to avoid and treat missing data

We have four strategies for limiting missing data in the conduct of this trial [28]: (1) selecting experienced investigators who have a good track record with respect to conducting clinical trials; (2) providing monetary incentives, which meet rigorous ethical requirement, to investigators and study staff for completeness of data collection; (3) providing reminder cards conveying the information that keeping participants in the trial until the end is important to investigators, study staff, and participants; and (4) keeping contact with participants. Missing data will be treated with the multiple imputation method.

### Power estimation and sample size calculation

As previously reported, the CARP (Coronary Artery Revascularization Prophylaxis) trial [13] shows a mortality of 23 % in the no-revascularization group, at a median of 2.7 years after randomization, and an incidence of MACE can be observed in 20 % of cases within 30 days after major vascular surgery. In addition, our previous study shows 25 % mortality, 8 % non-fatal myocardial infarction, 2 % non-fatal stroke, and 15 % coronary revascularization at a median of 10 months after PTA (total MACE rate is 35 %) [18]. Furthermore, a previous study showed that an absolute risk reduction of 16.7 % in MACE was observed in patients receiving routine coronary angiography versus selective group before major vascular surgery [6]. Therefore, we calculated that we would detect an absolute risk reduction of 16 % (estimated risk reduced from 40 % to 24 %) in primary composite events at long-term follow-up by enrolling a total sample of 450 patients (sample size of at least 432 with an estimated dropout rate of 4 %), with a one-sided alpha of 0.05, a power of 80 %, and a superior margin of 5 %. Although the superior margin is not absolutely necessary to demonstrate that the routine strategy is superior to the selective strategy, 5 % superior margin is to conservatively assess whether using the routine strategy would significantly reduce not only more but also at least 5 % greater in MACE than the selective strategy. The sample size would be smaller if there were no setup of the superior margin. Strategies for achieving adequate participant enrollment to reach the target sample size include selecting high-volume centers and interventionists for PTA and providing monetary incentives, which meet rigorous ethical requirements, to investigators and study staff for enrollment of participants.

### Statistical analysis plan

To compare baseline characteristics between routine and selective strategy groups as well as the routine strategy group and the registration group, all interval-scaled variables will be presented as mean ± standard deviation. Skewed interval-scaled data will be reported as median (interquartile range). Categorical variables will be presented as frequency and proportion (percentage). Chi-squared or Fisher’s exact test will be used for comparison of categorical variables between groups, whereas Mann-Whitney *U* test or unpaired Student’s *t* test will be used for interval-scaled variables as appropriate.

To compare the endpoints between routine and selective strategy groups, each analysis will be performed by “intention-to-treat” and “per protocol”. Proportional or Fisher’s exact test will be used for analysis of the proportion of primary, secondary, and other endpoints between routine and selective strategy groups as appropriate. For primary endpoint, significantly greater than 5 % reduction of MACE in the routine strategy group compared with the selective strategy group will be analyzed. By using the same statistical methods, subgroup analysis will include comparison of the proportion of endpoints in subgroups of these two randomized arms, such as patients with critical limb ischemia (Rutherford classification of at least 4), chronic kidney disease (at least stage 3), end-stage renal disease, old age, different genders, tobacco smoking, diabetes mellitus, hypertension, hyperlipidemia, metabolic syndrome, anemia, bedridden, hypoalbuminemia, elevated high-sensitivity C-reactive protein level, and so on. Kaplan-Meier analysis will be used to study patient survival and event-free status by using the log-rank test (Cox-Mantel) to ascertain differences between groups. If more than one endpoint occurs within the follow-up period for assessing event-free status, only the first event will be used in the analysis. Generalized estimating equation will be used to address whether and how the clustering by centers. Missing data will be treated with the multiple imputation method.

Although the main purpose of this study is not to compare the endpoints between the routine strategy group and the registration group or between randomized groups and the registration group, similar analyses as above will be performed to explore differences among patients in these groups. For all analyses, a *P* value of less than 0.05 is regarded as statistically significant. All statistical analyses are performed by using IBM SPSS Statistics 22.0 for Windows (IBM Corporation, Armonk, NY, USA), but the subgroup analysis is performed by using SAS software (Version 9.1.3; SAS Institute Inc., Cary, NC, USA).

## Discussion

The PIROUETTE-PTA study will be the first prospective RCT to determine the effectiveness of routine coronary angiography before PTA. Owing to the well-developed PCI technique and the innovation of excellent devices, contemporary PCI might provide not only symptom relief but also prognostic benefit [29]. If favorable, routine coronary angiography followed by revascularization may be a better coronary evaluation strategy with respect to cardiovascular outcomes in LEAD patients undergoing PTA.

All manuscripts and abstracts must be approved by the steering committee before they are submitted. The results will be disseminated via journal publication and abstract presentation on scientific conferences regardless of the magnitude or direction of effect. A summary of the study results will also be available at ClinicalTrials.gov. The publication plan of the study results for authorship policy and timeframe will be discussed and established before the last participant is enrolled.

### Trial status

The study is currently open for recruitment.

## Additional files

Additional file 1: Checklist of items for reporting trials of non-pharmacologic treatment. The information addressed in our study protocol complying with CONSORT extension for trials assessing non-pharmacologic intervention is provided in this checklist table. *CONSORT* Consolidated Standards of Reporting Trials. (PDF 613 kb) Additional file 2: SPIRIT 2013 Checklist: Recommended Items to Address in a Clinical Trial Protocol and Related Documents. The information addressed in our study protocol complying with SPIRIT 2013 is provided in this checklist table. *SPIRIT* Standard Protocol Items: Recommendations for Interventional Trials. (PDF 88 kb) Additional file 3: Case report forms (CRFs) of PIROUETTE-PTA Study. The file shows clinical data to be collected and recorded in 13 CRFs. *PIROUETTE-PTA* Prognostic Impact of Routine Coronary Catheterization in Low Extremity Artery Disease Undergoing Percutaneous Transluminal Angioplasty. (PDF 834 kb)

## Abbreviations

CAD: Coronary artery disease
CONSORT: Consolidated Standards of Reporting Trials
CRF: Case record form
CSIS: Clinical Study Information System
DSE: Dobutamine stress echocardiography
dTS: Dipyridamole thallium 201 myocardial perfusion scintigraphy
IRB: Institutional Review Board
LEAD: Low extremity artery disease
MACE: Composite major adverse cardiac event
PCI: Percutaneous coronary intervention
PIROUETTE-PTA: Prognostic Impact of Routine Coronary Catheterization in Low Extremity Artery Disease Undergoing Percutaneous Transluminal Angioplasty
PTA: Percutaneous transluminal angioplasty
RCT: Randomized controlled trial
SAE: Serious adverse event
