# Supplementary material for: Impact of routine coronary catheterization in low extremity artery disease undergoing percutaneous transluminal angioplasty: study protocol for a multi-center randomized controlled trial
Source: Trials. 2016 Feb 29;17:112. doi: 10.1186/s13063-016-1237-0 (PMC4772293; doi:10.1186/s13063-016-1237-0)
Supplement: Additional file 3: — Case report forms (CRFs) of PIROUETTE-PTA Study. The file shows clinical data to be collected and recorded in 13 CRFs. PIROUETTE-PTA Prognostic Impact of Routine Coronary Catheterization in Low Extremity Artery Disease Undergoing Percutaneous Transluminal Angioplasty. (PDF 834 kb) [file 13063_2016_1237_MOESM3_ESM.pdf]

**Case Report Form of PIROUETTE-PTA Study (Screening and Randomization)**

| |
|---|
| **Mark**：Yes : "v"、No : " –"、Not done: "ND"、Not applicable : "NA"<br>**Fill up every item.** |

Center ID: _________ Completion: Mandatory Date of form submission:_____

Patient ID: __________

Sex: ____________Age: _____________ Time of Screening: ________________

A. Inclusion: > 20 y/o with LEAD documented by angiography____(DSA___ ; CTA____; MRA_____) or sonography by operator______

B. Exclusion criteria: known CAD or unstable angina within past 3 months___; acute myocardial infarction within past 6 months____; known CAD status posterior PCI or bypass surgery within past 6 months____; planning to do bypass surgery for known LEAD____; pregnancy____; documented active malignancy____; needing emergency PTA____; withdrawal of consent_____

C. Grouping: routine____(fill J-1); selective____(fill J-2); registry_____

D. Underlying: DM_____(type 1___; type 2___) for ______years；insulin-used DM____；HTN_____；HL_____；Smoking____(quitted≥ 3yrs___; quitted< _______ 3yrs___; current___)；CAD_____；CAD S/P PCI_____；CAD S/P CABG_____；history of MI_____；history of CVA_____(ischemic_____；hemorrhagic_____；unidentified______)；S/P PTA____; S/P bypass for LEAD____；PUD_____；CKD_____(Stage_____)；ESRD_____for______ years；Non-ambulatory_____(totally bedridden____；wheelchair bound____)；

anemia_____；CHF_____(EF≥40%____；<40%____)；others_

E. ABI：Lt_____；Rt_____；# Fontaine class_____；Rutherford class_____

F. Symptoms/signs：No_____；Yes_____(intermittent claudication_____；leg swelling_____；cold foot_____；gangrene______; poor healing wound (ulcer)____; minor tissue loss____; major tissue loss____; others____)

G. Drugs used: Bokey_____；Plavix_____；Ticagrelor____; Ticocid_____；Pletaal_____; Trental_____；Persantin_____; ACEI_____；ARB_____；DRI_____; CCB_____(            ); Statin_____(           ); Fibrate_____(           ); TZD_____(           ); DPP4i_______(           ); GLP-1______(            ); others__ (specify_____)

H. Lab (on admission for cath or PTA as baseline lab data)

| | Albumin | AC sugar | BUN | Cr | Na | K | GPT | TC | TG | HDL | LDL | eGFR |
|---|---|---|---|---|---|---|---|---|---|---|---|---|
| unit | g/dL | mg/dL | | | mmol/L | | U/L | mg/dL | | | | mL/min/1.73m$^2$ |
| | | | | | | | | | | | | |

| Hb | WBC | PLT | N (Seg+Band) | L (lymphocytes) |
|---|---|---|---|---|
| g/dL | $10^3$/μl | | % | |
| | | | | |

I

| | BW (Kg) | BH (cm) | SBP (mmHg) | DBP (mmHg) | HR (/min) |
|---|---|---|---|---|---|
| | | | | | |

J.

1. Actually receiving coronary angiography: Yes____; No_____(why, specify_____)

2. Actually receiving stress test: Yes____; No_____(why, specify_____)

Signature: ______________________________ Date: __________________________

# Case Report Form of PIROUETTE-PTA Study (Cath)

**Mark**：Yes : ”v” 、No : “ –“ 、Not done: “ND” 、Not applicable : “NA”
**Fill up every item.**

Center ID: _________ Completion: in case Date of form submission:_________

Patient ID: __________

Sex: ____________Age: _____________ Time of Screening: _________________

A. Date of coronary angiography_________; Time elapsed from screening: _______ days; Indications: routine____; positive stress______(DSE_____; dTS______; TXT____); symptoms suggestive of angina____(stable___; unstable____; MI____); EKG suggestive of CAD____; cardiac enzymes suggestive of ACS____; Holter suggestive of ischemia____; cardiac echo suggestive of CAD____; MSCTA suggestive of CAD____

B. Results of coronary angiography: normal___; non-obstructive atherosclerosis___; CAD___

C. If tick CAD in “B”: LM____; 4VD____; 3VD____; 2VD____; 1VD_____; Syntax score_____

D. Vessels involved (if tick CAD in “B”): LM___; LAD___ ; LCX____; RCA____; IM____; arterial graft____ (LIMA____ to which vessel _____; RIMA____ to which vessel _____ ; others, specify____ to which vessel_____) ; venous graft___ (SVG1____ to which vessel_____; SVG2____ to which vessel____ ; SVG3____ to which vessel _____ ; others, specify____ to which vessel_____)

E. Complications of coronary angiography: No____; Yes____(specify:___________)

F. Immediate outcomes of coronary angiography: No event____; Events____
(non-fatal MI___; non-fatal stroke____; cardiovascular death____ [fatal MI____; fatal stroke____] ; cardiogenic shock_____; sudden cardiac death_____] ; total death______[specified _______________________________________________])

G. Recommendation: Revascularization_____(PCI_____; CABG_____); Medical treatment ______

H. Final: Revascularization_____(PCI_____[fill form CRF003]; CABG_____[fill form CRF004]); Medical treatment ______)

I. Adjunct tools to facilitate confirmation of coronary severity: FFR____ ; IVUS____; OCT____

Signature: ____________________________ Date: _________________________

# Case Report Form of PIROUETTE-PTA Study (PCI)

| |
|---|
| **Mark**：Yes : ”v” 、No : “ –“ 、Not done: “ND” 、Not applicable : “NA”<br>**Fill up every item.** |

Center ID: _________ Completion: in case Date of form submission:_________

Patient ID: __________

Sex: ____________Age: _____________ Time of Screening: _________________

A. Date of PCI______________; Time elapsed from screening: _______days

B. How many vessels are intervened?: LM____; 4_____; 3____; 2____; 1_____

C. Which vessels are intervened?:

1. LM___(POBA___; BMS___[Multi-Link___; Integrity___; others, specify____]; DES___[Xience Prime____; Resolute Integrity____; others, specify____])
2. LAD___(POBA___; BMS___[Multi-Link___; Integrity___; others, specify____]; DES___[Xience Prime____; Resolute Integrity____; others, specify____])
3. LCX____(POBA___; BMS___[Multi-Link___; Integrity___; others, specify____]; DES___[Xience Prime____; Resolute Integrity____; others, specify____])
4. RCA____(POBA___; BMS___[Multi-Link___; Integrity___; others, specify____]; DES___[Xience Prime____; Resolute Integrity____; others, specify____])
5. IM____(POBA___; BMS___[Multi-Link___; Integrity___; others, specify____]; DES___[Xience Prime____; Resolute Integrity____; others, specify____])

6. Artery graft____(LIMA____; RIMA____; others, specify____) (POBA___; BMS___[Multi-Link___; Integrity___; others, specify____]; DES___[Xience Prime____; Resolute Integrity____; others, specify____])

7. Venous graft____(SVG1_____; SVG2_____; SVG3_____; others, specify____) (POBA___; BMS___[Multi-Link___; Integrity___; others, specify____]; DES_ [Xience Prime____; Resolute Integrity____; others, specify____])

D. How many lesions are intervened?:_______

E. How many stents are deployed? _________

F. Complications of PCI: No____; Yes____(specify:__________________________)

G. Immediate outcomes of coronary angiography: No event____; Events____ (non-fatal MI___; non-fatal stroke____; cardiovascular death____ [fatal MI____; fatal stroke____; cardiogenic shock_____; sudden cardiac death_____] ; total death______[specified ______________________________________________])

H. Results of PCI: Success_____(complete_____; partial_____); failure ______

I. Cross over to CABG: No_____; Yes____(fill form CRF004)

Signature: ____________________________ Date: _________________________

# Case Report Form of PIROUETTE-PTA Study (CABG)

Center ID: _________ Completion: in case Date of form submission:_________

Patient ID: __________

Sex: ____________Age: _____________ Time of Screening: __________________

A. Date of CABG______________; Time elapsed from screening: _______days

B. Type: Elective______; Urgency or Emergency_______

C. Beating heart_______; non-beating heart ______

D. How many vessels are bypassed?: 4_____; 3____; 2____; 1_____

E. Which vessels are bypassed?:

1. LAD___(vein graft___; artery graft____)
2. LCX____(vein graft___; artery graft____)
3. RCA____(vein graft___; artery graft____)
4. IM____(vein graft___; artery graft____)

F. How many grafts are implanted?:_______

G. Complications of CABG: No____; Yes____

H. Immediate outcomes of CABG: No event____; Events____(non-fatal MI___;

non-fatal stroke____; cardiovascular death____ [fatal MI____; fatal stroke____] ;

cardiogenic shock_____; sudden cardiac death_____] ; total death______

[specified ____________________________________________])

I. Results of CABG: Success_____; failure ______

J. Cross over to PCI: No_____; Yes____(fill form CRF003)

K. Duration of hospitalization:__________days

Signature: ____________________________ Date: _________________________

# Case Report Form of PIROUETTE-PTA Study (PTA)

**Mark**：Yes : "v"、No : " –"、Not done: "ND"、Not applicable : "NA"
**Fill up every item.**

Center ID: _________ Completion: Mandatory Date of form submission:_______

Patient ID: __________

Sex: ____________Age: _____________ Time of Screening: __________________

A. Date of PTA_________; Time elapsed from screening: _______days; Time elapsed from revascularization (PCI or CABG) _________days; previous ipsilateral PTA_______; previous contralateral PTA______; previous ipsilateral bypass_______; previous contralateral bypass______

B. **Angiographic characteristics [TASC-II type sees J Vasc Surg 2007;45(supl S):S1-S67]**

1. Rt lesions：Iliac_____(_____% stenosis；calcification_____；lesion length___ mm；reference vessel size_____mm；collateral_____；TASC-II type_____)；Femopopliteal_____(_____% stenosis；calcification_____；lesion length_____ mm；reference vessel size_____mm；collateral_____；TASC-II type_____)；Infrapopliteal_____(____VD；ATA_____[_____% stenosis；calcification_____；lesion length_____mm；reference vessel size_____mm；collateral_____]；PTA_____[_____% stenosis；calcification_____；lesion length mm；reference vessel size_____mm；collateral_____]；peroneal_____[_____% stenosis；calcification_____；lesion length_____mm；reference vessel size___ mm；collateral_____]；number of tibial run-off______)

2. Lt lesions：Iliac_____(_____% stenosis；calcification_____；lesion length___

mm；reference vessel size_____mm；collateral_____；TASC-II type_____)；

Femopopliteal_____(_____% stenosis；calcification_____；lesion length_____ mm；reference vessel size_____mm；collateral_____；TASC-II type_____)；

Infrapopliteal_____(____VD；ATA_____[_____% stenosis；calcification_____；lesion length_____mm；reference vessel size_____mm；collateral_____]；PTA_____[_____% stenosis；calcification_____；lesion length mm；reference vessel size_____mm；collateral_____]；peroneal_____[_____% stenosis；calcification_____；lesion length_____mm；reference vessel size___ mm；collateral_____]；number of tibial run-off______)

3. Right digital circulation：Grade_____

Left digital circulation：Grade_____

(P.S.：Grade 0：no digital circulation；Grade 1：circulating flow to less than 5 toes or inadequate flow to 5 toes；Grade 2: adequate flow to 5 toes)

4. Classification of pedal arch：

Right：type 1____；type 2A____；type 2B____；type 3_____

Left：type 1____；type 2A____；type 2B____；type 3_____

(P.S.：type 1：both dorsalis pedis and plantar arteries patent；type 2A：only dorsalis pedis patent；type 2B：only plantar artery patent；type 3：both dorsalis pedis and plantar arteries occluded)

**C. Angioplasty parameters**

1. Approach methods：

a. Antegrade_____：transfemoral_____(contralateral antegrade_____；ipsilateral antegrade_____)；transbrachial antegrade_____

b. Retrograde_____：distal SFA______；popliteal______；distal PTA______；

dorsal pedis______ ; others______[specify:__________________________])

2. Special devices used : laser______; re-entry device_______(off-road_____; outback______; others______[specify_______]); rotablation______ ; cutting balloon______; distal embolism protection device_______ ; others______ (specify: _________________________)

3. Methods for CTO lesions : subintimal tracking______ ; Re-entry device______ ; double-balloon technique______; SAFARI technique______ ; others______ (specify: _______________)

4. PTA for ilac_____(Left______ ; Right_______)

POBA______(size : ______mm ; length : ______mm ; Brand : __________) ;

DEB______(size : ______mm ; length : ______mm ; Brand : __________) ;

Stenting: Yes_____(provisional_____; routine_____); No_____

BMS______(size : ______mm ; length : ______mm ; Self-expandable stent______ ; balloon-expandable stent______; Brand : __________)

DES______(size : ______mm ; length : ______mm ; Self-expandable stent______ ; balloon-expandable stent______; Brand : __________)

5. PTA for CFA or SFA_____(Left______ ; Right_______)

POBA______(size : ______mm ; length : ______mm ; Brand : __________) ;

DEB______(size : ______mm ; length : ______mm ; Brand : __________) ;

Stenting: Yes_____(provisional_____; routine_____); No_____

BMS______(size : ______mm ; length : ______mm ; Self-expandable stent______ ; balloon-expandable stent______; Brand : __________)

DES______(size : ______mm ; length : ______mm ; Self-expandable stent______ ; balloon-expandable stent______; Brand : __________)

6. PTA for popliteal_____(Left______ ; Right_______)

POBA______(size：______mm；length：______mm；Brand：__________)；

DEB______(size：______mm；length：______mm；Brand：__________)；

Stenting: Yes_____(provisional_____; routine_____); No_____

BMS______(size：______mm；length：______mm；Self-expandable

stent______；balloon-expandable stent______; Brand：__________)

DES______(size：______mm；length：______mm；Self-expandable

stent______；balloon-expandable stent______; Brand：__________)

7. PTA for infrapopliteal_____(Left______；Right_______)

(1) PTA for ATA_______(Left______；Right_______)

POBA______(size：______mm；length：______mm；Brand：__________)；

DEB______(size：______mm；length：______mm；Brand：__________)；

Stenting: Yes_____(provisional_____; routine_____); No_____

BMS______(size：______mm；length：______mm；Self-expandable

stent______；balloon-expandable stent______; Brand：__________)

DES______(size：______mm；length：______mm；Self-expandable

stent______；balloon-expandable stent______; Brand：__________)

(2) PTA for PTA_______(Left______；Right_______)

POBA______(size：______mm；length：______mm；Brand：__________)；

DEB______(size：______mm；length：______mm；Brand：__________)；

Stenting: Yes_____(provisional_____; routine_____); No_____

BMS______(size：______mm；length：______mm；Self-expandable

stent______；balloon-expandable stent______; Brand：__________)

DES______(size：______mm；length：______mm；Self-expandable

stent______；balloon-expandable stent______; Brand：__________)

(3) PTA for peroneal artery_______(Left______；Right_______)

POBA______(size：______mm；length：______mm；Brand：__________)；

DEB______(size：______mm；length：______mm；Brand：__________)；

Stenting: Yes_____(provisional_____; routine_____); No_____

BMS______(size：______mm；length：______mm；Self-expandable stent______；balloon-expandable stent______; Brand：__________)

DES______(size：______mm；length：______mm；Self-expandable stent______；balloon-expandable stent______; Brand：__________)

8. For SFA lesion, combined inflow intervention (iliac or CFA)______; combined outflow intervention (infrapopliteal)______

9. Complications：residual dissection______；thrombus formation______；dislodged stent______；migrating stent with resultant uncovered lesion______；vessel rupture______；vascular access complications______；shock______；distal embolism_____；acute occlusion______；others______ [specify:__________________________________])

10. Immediate outcome：complete success______；partial success_____；failure_____

11. If tick "complete success" or "partial success" in question 10:

Angiosome success_________; boundary success__________

12. After PTA,

Right digital circulation：Grade_____

Left digital circulation：Grade_____

(P.S.：Grade 0：no digital circulation；Grade 1：circulating flow to less than 5 toes or inadequate flow to 5 toes；Grade 2: adequate flow to 5 toes)

13. After PTA, pedal arch classification：

Right：type 1____；type 2A____；type 2B____；type 3_____

Left：type 1____；type 2A____；type 2B____；type 3_____

(P.S.：type 1：both dorsalis pedis and plantar arteries patent；type 2A：only dorsalis pedis patent；type 2B：only plantar artery patent；type 3：both dorsalis pedis and plantar arteries occluded)

14. After PTA, wound brush (if there is an unhealed wound before PTA) will be

Right：No______; Yes ______

Left：No______; Yes ______

15. Immediate CV outcomes of PTA: No event____; Events____(non-fatal MI___; non-fatal stroke____; cardiovascular death____ [fatal MI____; fatal stroke____; cardiogenic shock_____; sudden cardiac death_____] ; total death______ [specified ______________________________________________])

16. Cross over to bypass surgery: No_____; Yes______(why, specific__________)

17. Procedure time:_____min; Fluoro time:_______min; Contrast used:______ml

Signature: ______________________________ Date: ________________________

# Case Report Form of PIROUETTE-PTA Study (Visit 1)

**Mark**：Yes : ”v”、No : “ –“、Not done: “ND”、Not applicable : “NA”
**Fill up every item.**

Center ID: _________ Completion: Mandatory Date of form submission:_______

Patient ID: __________

Sex: ____________Age: _____________ Time of Screening: _________________

A. ABI：Lt_____；Rt_____；# Fontaine class_____；Rutherford class_____

B. Symptoms/signs：No_____；Yes_____(intermittent claudication_____；leg swelling_____；cold foot_____；gangrene______; poor healing wound (ulcer)____; minor tissue loss____; major tissue loss____; others____)

C. Drugs used: Bokey_____；Plavix_____；Ticagrelor____; Ticocid_____；Pletaal_____; Trental_____；Persantin_____; ACEI_____；ARB_____；DRI ; CCB_____( ); Statin_____( ); Fibrate_____( ); TZD_____ ( ); DPP4i_______( ); GLP-1______( ); others (specified )

D.

| | Date of occurrence | Time elapse from Screening (days) | Is it planned staged PCI (or CABG) or PTA (or bypass) | Is it related to cath, PCI or CABG? |
|---|---|---|---|---|
| Symptom-free | | | NA | NA |
| Intermittent | | | NA | NA |

| | | | | |
|---|---|---|---|---|
| claudication | | | | |
| Resting pain or numbness | | | NA | NA |
| Ulcer wound | | | NA | NA |
| Gangrene | | | NA | NA |
| Minor amputation | | | NA | NA |
| Major amputation | | | NA | NA |
| Total Death | | | NA | No____; Yes__ (cath_____; PCI_____; CABG_____) Cause of death: ____________ |
| CV death | | | NA | No____; Yes__ (cath_____; PCI_____; CABG_____) |
| Cardiogenic shock | | | NA | No____; Yes__ (cath_____; PCI_____; CABG_____) |
| Sudden cardiac death | | | NA | No____; Yes__ (cath_____; PCI_____; CABG_____) |
| Fatal stroke | | | NA | No____; Yes__ (cath_____; PCI_____; CABG_____) |
| Fatal MI | | | NA | No____; Yes__ |

| | | | | |
|---|---|---|---|---|
| | | | | (cath_____; PCI_____; CABG_____) |
| Non-fatal MI | | | NA | No____; Yes__ (cath_____; PCI_____; CABG_____) |
| Non-fatal stroke | | | NA | No____; Yes__ (cath_____; PCI_____; CABG_____) |
| CHF hospitalization | | | NA | No____; Yes__ (cath_____; PCI_____; CABG_____) |
| New ESRD (in pts with no ESRD at baseline) | | | NA | No____; Yes__ (cath_____; PCI_____; CABG_____) |
| TLR (PAD) | | | No____ (ischemia-driven___ [symptoms____]; based on images___ [duplex____; ABI____; CTA____; MRA____; DSA____]); Yes___ | NA |
| TVR (PAD) | | | No____ (ischemia-driven___ [symptoms____]; based on images___ [duplex____; | NA |

| | | | | |
|---|---|---|---|---|
| | | | ABI____; CTA____; MRA____; DSA____]); Yes___ | |
| TLR (CAD) | | | No____ (ischemia-driven___ [symptoms______; stress test______]; based on images___ [CTA____; MRA____; cath____]); Yes____ | NA |
| TVR (CAD) | | | No____ (ischemia-driven___ [symptoms______; stress test______]; based on images___ [CTA____; MRA____; cath____]); Yes____ | NA |
| Any Revascularization for PAD | | | No____ (ischemia-driven___ [symptoms____]; based on images___ [duplex____; ABI____; CTA____; MRA____; DSA____]); Yes___ | NA |
| Any revascularization for CAD | | | No____ (ischemia-driven___ [symptoms______; stress test______]; | NA |

| | | | | |
|---|---|---|---|---|
| | | | based on images____ [CTA____; MRA____; cath____]); Yes____ | |
| Recurrent angina | | | NA | NA |

Signature: ______________________________ Date: _________________________

# Case Report Form of PIROUETTE-PTA Study (Visit 2)

**Mark**：Yes : ”v” 、No : “ –“ 、Not done: “ND” 、Not applicable : “NA”
**Fill up every item.**

Center ID: _________ Completion: Mandatory Date of form submission:_______

Patient ID: __________

Sex: ____________Age: _____________ Time of Screening: _________________

A. ABI：Lt_____；Rt_____；# Fontaine class_____；Rutherford class_____

B. Symptoms/signs：No_____；Yes_____(intermittent claudication_____；leg swelling_____；cold foot_____；gangrene______; poor healing wound (ulcer)____; minor tissue loss____; major tissue loss____; others____)

C. Drugs used: Bokey_____；Plavix_____；Ticagrelor____; Ticocid_____；Pletaal_____; Trental_____；Persantin_____; ACEI_____；ARB_____；DRI ; CCB_____( ); Statin_____( ); Fibrate_____( ); TZD_____ ( ); DPP4i_______( ); GLP-1______( ); others (specified )

D.

| | Date of occurrence | Time elapse from Screening (days) | Is it planned staged PCI (or CABG) or PTA (or bypass) | Is it related to cath, PCI or CABG? |
|---|---|---|---|---|
| Symptom-free | | | NA | NA |
| Intermittent | | | NA | NA |

| | | | | |
|---|---|---|---|---|
| claudication | | | | |
| Resting pain or numbness | | | NA | NA |
| Ulcer wound | | | NA | NA |
| Gangrene | | | NA | NA |
| Minor amputation | | | NA | NA |
| Major amputation | | | NA | NA |
| Total Death | | | NA | No____; Yes__ (cath_____; PCI_____; CABG_____) Cause of death: ____________ |
| CV death | | | NA | No____; Yes__ (cath_____; PCI_____; CABG_____) |
| Cardiogenic shock | | | NA | No____; Yes__ (cath_____; PCI_____; CABG_____) |
| Sudden cardiac death | | | NA | No____; Yes__ (cath_____; PCI_____; CABG_____) |
| Fatal stroke | | | NA | No____; Yes__ (cath_____; PCI_____; CABG_____) |
| Fatal MI | | | NA | No____; Yes__ |

| | | | | |
|---|---|---|---|---|
| | | | | (cath_____; PCI_____; CABG_____) |
| Non-fatal MI | | | NA | No____; Yes__ (cath_____; PCI_____; CABG_____) |
| Non-fatal stroke | | | NA | No____; Yes__ (cath_____; PCI_____; CABG_____) |
| CHF hospitalization | | | NA | No____; Yes__ (cath_____; PCI_____; CABG_____) |
| New ESRD (in pts with no ESRD at baseline) | | | NA | No____; Yes__ (cath_____; PCI_____; CABG_____) |
| TLR (PAD) | | | No____ (ischemia-driven___ [symptoms____]; based on images___ [duplex____; ABI____; CTA____; MRA____; DSA____]); Yes___ | NA |
| TVR (PAD) | | | No____ (ischemia-driven___ [symptoms____]; based on images___ [duplex____; | NA |

| | | | | |
|---|---|---|---|---|
| | | | ABI____;<br>CTA____;<br>MRA____;<br>DSA____]); Yes___ | |
| TLR (CAD) | | | No____<br>(ischemia-driven___<br>[symptoms______;<br>stress test______];<br>based on images___<br>[CTA____;<br>MRA____;<br>cath____]); Yes____ | NA |
| TVR (CAD) | | | No____<br>(ischemia-driven___<br>[symptoms______;<br>stress test______];<br>based on images___<br>[CTA____;<br>MRA____;<br>cath____]); Yes____ | NA |
| Any Revascularization for PAD | | | No____<br>(ischemia-driven___<br>[symptoms____];<br>based on images___<br>[duplex____;<br>ABI____;<br>CTA____;<br>MRA____;<br>DSA____]); Yes___ | NA |
| Any revascularization for CAD | | | No____<br>(ischemia-driven___<br>[symptoms______;<br>stress test______]; | NA |

| | | | | |
|---|---|---|---|---|
| | | | based on images____ [CTA____; MRA____; cath____]); Yes____ | |
| Recurrent angina | | | NA | NA |

Signature: ______________________________ Date: _________________________

## Case Report Form of PIROUETTE-PTA Study (Visit 3)

**Mark**：Yes : ”v”、No : “ –“、Not done: “ND”、Not applicable : “NA”
**Fill up every item.**

Center ID: _________ Completion: Mandatory Date of form submission:_______

Patient ID: __________

Sex: ____________Age: _____________ Time of Screening: __________________

A. ABI：Lt_____；Rt_____；# Fontaine class_____；Rutherford class_____

B. Symptoms/signs：No_____；Yes_____(intermittent claudication_____；leg swelling_____；cold foot_____；gangrene______; poor healing wound (ulcer)____; minor tissue loss____; major tissue loss____; others____)

C. Drugs used: Bokey_____；Plavix_____；Ticagrelor____; Ticocid_____；Pletaal_____; Trental_____；Persantin_____; ACEI_____；ARB_____；DRI ; CCB_____( ); Statin_____( ); Fibrate_____( ); TZD_____ ( ); DPP4i_______( ); GLP-1______( ); others (specified )

D.

| | Date of occurrence | Time elapse from Screening (days) | Is it planned staged PCI (or CABG) or PTA (or bypass) | Remarks |
|---|---|---|---|---|
| Symptom-free | | | NA | |
| Intermittent | | | NA | |

| | | | | |
|---|---|---|---|---|
| claudication | | | | |
| Resting pain or numbness | | | NA | |
| Ulcer wound | | | NA | |
| Gangrene | | | NA | |
| Minor amputation | | | NA | |
| Major amputation | | | NA | |
| Total Death | | | NA | |
| CV death | | | NA | |
| Cardiogenic shock | | | NA | |
| Sudden cardiac death | | | NA | |
| Fatal stroke | | | NA | |
| Fatal MI | | | NA | |
| Non-fatal MI | | | NA | |
| Non-fatal stroke | | | NA | |
| CHF hospitalization | | | NA | |
| New ESRD (in pts with no ESRD at baseline) | | | NA | |
| TLR (PAD) | | | No____ (ischemia-driven____ [symptoms____]; | |

| | | | | |
|---|---|---|---|---|
| | | | based on images____ [duplex____; ABI____; CTA____; MRA____; DSA____]); Yes____ | |
| TVR (PAD) | | | No____ (ischemia-driven____ [symptoms____]; based on images____ [duplex____; ABI____; CTA____; MRA____; DSA____]); Yes____ | |
| TLR (CAD) | | | No____ (ischemia-driven____ [symptoms______; stress test______]; based on images____ [CTA____; MRA____; cath____]); Yes____ | |
| TVR (CAD) | | | No____ (ischemia-driven____ [symptoms______; stress test______]; based on images____ [CTA____; MRA____; cath____]); Yes____ | |
| Any Revascularization for PAD | | | No____ (ischemia-driven____ [symptoms____]; based on images____ | |

| | | | | |
|---|---|---|---|---|
| | | | [duplex____; ABI____; CTA____; MRA____; DSA____]); Yes____ | |
| Any revascularization for CAD | | | No____ (ischemia-driven____ [symptoms______; stress test______]; based on images____ [CTA____; MRA____; cath____]); Yes____ | |
| Recurrent angina | | | NA | |

Signature: ______________________________ Date: _________________________

## Case Report Form of PIROUETTE-PTA Study (Visit 4)

**Mark**：Yes : ”v” 、No : “ –“ 、Not done: “ND” 、Not applicable : “NA”
**Fill up every item.**

Center ID: _________ Completion: Mandatory Date of form submission:_______

Patient ID: __________

Sex: ____________Age: _____________ Time of Screening: __________________

A. ABI：Lt_____ ；Rt_____ ；# Fontaine class_____ ；Rutherford class_____

B. Symptoms/signs：No_____ ；Yes_____(intermittent claudication_____ ；leg swelling_____；cold foot_____；gangrene______; poor healing wound (ulcer)____; minor tissue loss____; major tissue loss____; others____)

C. Drugs used: Bokey_____ ；Plavix_____ ；Ticagrelor____; Ticocid_____ ； Pletaal_____; Trental_____；Persantin_____; ACEI_____；ARB_____；DRI ; CCB_____( ); Statin_____( ); Fibrate_____( ); TZD_____ ( ); DPP4i_______( ); GLP-1______( ); others (specified )

D.

| | Date of occurrence | Time elapse from Screening (days) | Is it planned staged PCI (or CABG) or PTA (or bypass) | Remarks |
|---|---|---|---|---|
| Symptom-free | | | NA | |
| Intermittent | | | NA | |

| | | | | |
|---|---|---|---|---|
| claudication | | | | |
| Resting pain or numbness | | | NA | |
| Ulcer wound | | | NA | |
| Gangrene | | | NA | |
| Minor amputation | | | NA | |
| Major amputation | | | NA | |
| Total Death | | | NA | |
| CV death | | | NA | |
| Cardiogenic shock | | | NA | |
| Sudden cardiac death | | | NA | |
| Fatal stroke | | | NA | |
| Fatal MI | | | NA | |
| Non-fatal MI | | | NA | |
| Non-fatal stroke | | | NA | |
| CHF hospitalization | | | NA | |
| New ESRD (in pts with no ESRD at baseline) | | | NA | |
| TLR (PAD) | | | No____ (ischemia-driven____ [symptoms____]; | |

| | | | | |
|---|---|---|---|---|
| | | | based on images____ [duplex____; ABI____; CTA____; MRA____; DSA____]); Yes____ | |
| TVR (PAD) | | | No____ (ischemia-driven____ [symptoms____]; based on images____ [duplex____; ABI____; CTA____; MRA____; DSA____]); Yes____ | |
| TLR (CAD) | | | No____ (ischemia-driven____ [symptoms______; stress test______]; based on images____ [CTA____; MRA____; cath____]); Yes____ | |
| TVR (CAD) | | | No____ (ischemia-driven____ [symptoms______; stress test______]; based on images____ [CTA____; MRA____; cath____]); Yes____ | |
| Any Revascularization for PAD | | | No____ (ischemia-driven____ [symptoms____]; based on images____ | |

| | | | | |
|---|---|---|---|---|
| | | | [duplex____; ABI____; CTA____; MRA____; DSA____]); Yes____ | |
| Any revascularization for CAD | | | No____ (ischemia-driven____ [symptoms______; stress test______]; based on images____ [CTA____; MRA____; cath____]); Yes____ | |
| Recurrent angina | | | NA | |

Signature: ______________________________ Date: _________________________

# Case Report Form of PIROUETTE-PTA Study (Visit 5)

**Mark**：Yes : ”v”、No : “ –“、Not done: “ND”、Not applicable : “NA”
**Fill up every item.**

Center ID: _________ Completion: Mandatory Date of form submission:_______

Patient ID: __________

Sex: ____________Age: _____________ Time of Screening: __________________

A. ABI：Lt_____；Rt_____；# Fontaine class_____；Rutherford class_____

B. Symptoms/signs：No_____；Yes_____(intermittent claudication_____；leg swelling_____；cold foot_____；gangrene______; poor healing wound (ulcer)____; minor tissue loss____; major tissue loss____; others____)

C. Drugs used: Bokey_____；Plavix_____；Ticagrelor____; Ticocid_____；Pletaal_____; Trental_____；Persantin_____; ACEI_____；ARB_____；DRI ; CCB_____( ); Statin_____( ); Fibrate_____( ); TZD_____ ( ); DPP4i_______( ); GLP-1______( ); others (specified )

D.

| | Date of occurrence | Time elapse from Screening (days) | Is it planned staged PCI (or CABG) or PTA (or bypass) | Remarks |
|---|---|---|---|---|
| Symptom-free | | | NA | |
| Intermittent | | | NA | |

| | | | | |
|---|---|---|---|---|
| claudication | | | | |
| Resting pain or numbness | | | NA | |
| Ulcer wound | | | NA | |
| Gangrene | | | NA | |
| Minor amputation | | | NA | |
| Major amputation | | | NA | |
| Total Death | | | NA | |
| CV death | | | NA | |
| Cardiogenic shock | | | NA | |
| Sudden cardiac death | | | NA | |
| Fatal stroke | | | NA | |
| Fatal MI | | | NA | |
| Non-fatal MI | | | NA | |
| Non-fatal stroke | | | NA | |
| CHF hospitalization | | | NA | |
| New ESRD (in pts with no ESRD at baseline) | | | NA | |
| TLR (PAD) | | | No____ (ischemia-driven____ [symptoms____]; | |

| | | | | |
|---|---|---|---|---|
| | | | based on images____ [duplex____; ABI____; CTA____; MRA____; DSA____]); Yes____ | |
| TVR (PAD) | | | No____ (ischemia-driven____ [symptoms____]; based on images____ [duplex____; ABI____; CTA____; MRA____; DSA____]); Yes____ | |
| TLR (CAD) | | | No____ (ischemia-driven____ [symptoms______; stress test______]; based on images____ [CTA____; MRA____; cath____]); Yes____ | |
| TVR (CAD) | | | No____ (ischemia-driven____ [symptoms______; stress test______]; based on images____ [CTA____; MRA____; cath____]); Yes____ | |
| Any Revascularization for PAD | | | No____ (ischemia-driven____ [symptoms____]; based on images____ | |

| | | | | |
|---|---|---|---|---|
| | | | [duplex____; ABI____; CTA____; MRA____; DSA____]); Yes____ | |
| Any revascularization for CAD | | | No____ (ischemia-driven____ [symptoms______; stress test______]; based on images____ [CTA____; MRA____; cath____]); Yes____ | |
| Recurrent angina | | | NA | |

Signature: ______________________________ Date: _________________________

## Case Report Form of PIROUETTE-PTA Study (Visit 6)

**Mark**：Yes : "v" 、No : " –" 、Not done: "ND" 、Not applicable : "NA"
**Fill up every item.**

Center ID: _________ Completion: Mandatory Date of form submission:_______

Patient ID: __________

Sex: ____________Age: _____________ Time of Screening: __________________

A. ABI：Lt_____ ；Rt_____ ；# Fontaine class_____ ；Rutherford class_____

B. Symptoms/signs：No_____ ；Yes_____(intermittent claudication_____ ；leg swelling_____；cold foot_____；gangrene______; poor healing wound (ulcer)____; minor tissue loss____; major tissue loss____; others____)

C. Drugs used: Bokey_____ ；Plavix_____ ；Ticagrelor____; Ticocid_____ ； Pletaal_____; Trental_____；Persantin_____; ACEI_____；ARB_____；DRI ; CCB_____( ); Statin_____( ); Fibrate_____( ); TZD_____ ( ); DPP4i_______( ); GLP-1______( ); others (specified )

D.

| | Date of occurrence | Time elapse from Screening (days) | Is it planned staged PCI (or CABG) or PTA (or bypass) | Remarks |
|---|---|---|---|---|
| Symptom-free | | | NA | |
| Intermittent | | | NA | |

| | | | | |
|---|---|---|---|---|
| claudication | | | | |
| Resting pain or numbness | | | NA | |
| Ulcer wound | | | NA | |
| Gangrene | | | NA | |
| Minor amputation | | | NA | |
| Major amputation | | | NA | |
| Total Death | | | NA | |
| CV death | | | NA | |
| Cardiogenic shock | | | NA | |
| Sudden cardiac death | | | NA | |
| Fatal stroke | | | NA | |
| Fatal MI | | | NA | |
| Non-fatal MI | | | NA | |
| Non-fatal stroke | | | NA | |
| CHF hospitalization | | | NA | |
| New ESRD (in pts with no ESRD at baseline) | | | NA | |
| TLR (PAD) | | | No____ (ischemia-driven____ [symptoms____]; | |

| | | | | |
|---|---|---|---|---|
| | | | based on images____ [duplex____; ABI____; CTA____; MRA____; DSA____]); Yes____ | |
| TVR (PAD) | | | No____ (ischemia-driven____ [symptoms____]; based on images____ [duplex____; ABI____; CTA____; MRA____; DSA____]); Yes____ | |
| TLR (CAD) | | | No____ (ischemia-driven____ [symptoms______; stress test______]; based on images____ [CTA____; MRA____; cath____]); Yes____ | |
| TVR (CAD) | | | No____ (ischemia-driven____ [symptoms______; stress test______]; based on images____ [CTA____; MRA____; cath____]); Yes____ | |
| Any Revascularization for PAD | | | No____ (ischemia-driven____ [symptoms____]; based on images____ | |

| | | | | |
|---|---|---|---|---|
| | | | [duplex____; ABI____; CTA____; MRA____; DSA____]); Yes____ | |
| Any revascularization for CAD | | | No____ (ischemia-driven____ [symptoms______; stress test______]; based on images____ [CTA____; MRA____; cath____]); Yes____ | |
| Recurrent angina | | | NA | |

Signature: ______________________________ Date: _________________________

# Case Report Form of PIROUETTE-PTA Study (Visit 7)

**Mark**：Yes : "v"、No : " –"、Not done: "ND"、Not applicable : "NA"
**Fill up every item.**

Center ID: _________ Completion: Mandatory Date of form submission:_______

Patient ID: __________

Sex: ____________Age: _____________ Time of Screening: __________________

A. ABI：Lt_____ ；Rt_____ ；# Fontaine class_____ ；Rutherford class_____

B. Symptoms/signs：No_____ ；Yes_____(intermittent claudication_____ ；leg swelling_____；cold foot_____；gangrene______; poor healing wound (ulcer)____; minor tissue loss____; major tissue loss____; others____)

C. Drugs used: Bokey_____ ；Plavix_____ ；Ticagrelor____; Ticocid_____ ；Pletaal_____; Trental_____；Persantin_____; ACEI_____；ARB_____；DRI ; CCB_____( ); Statin_____( ); Fibrate_____( ); TZD_____ ( ); DPP4i_______( ); GLP-1______( ); others (specified )

D.

| | Date of occurrence | Time elapse from Screening (days) | Is it planned staged PCI (or CABG) or PTA (or bypass) | Remarks |
|---|---|---|---|---|
| Symptom-free | | | NA | |
| Intermittent | | | NA | |

| | | | | |
|---|---|---|---|---|
| claudication | | | | |
| Resting pain or numbness | | | NA | |
| Ulcer wound | | | NA | |
| Gangrene | | | NA | |
| Minor amputation | | | NA | |
| Major amputation | | | NA | |
| Total Death | | | NA | |
| CV death | | | NA | |
| Cardiogenic shock | | | NA | |
| Sudden cardiac death | | | NA | |
| Fatal stroke | | | NA | |
| Fatal MI | | | NA | |
| Non-fatal MI | | | NA | |
| Non-fatal stroke | | | NA | |
| CHF hospitalization | | | NA | |
| New ESRD (in pts with no ESRD at baseline) | | | NA | |
| TLR (PAD) | | | No____ (ischemia-driven____ [symptoms____]; | |

| | | | | |
|---|---|---|---|---|
| | | | based on images____ [duplex____; ABI____; CTA____; MRA____; DSA____]); Yes____ | |
| TVR (PAD) | | | No____ (ischemia-driven____ [symptoms____]; based on images____ [duplex____; ABI____; CTA____; MRA____; DSA____]); Yes____ | |
| TLR (CAD) | | | No____ (ischemia-driven____ [symptoms______; stress test______]; based on images____ [CTA____; MRA____; cath____]); Yes____ | |
| TVR (CAD) | | | No____ (ischemia-driven____ [symptoms______; stress test______]; based on images____ [CTA____; MRA____; cath____]); Yes____ | |
| Any Revascularization for PAD | | | No____ (ischemia-driven____ [symptoms____]; based on images____ | |

| | | | | |
|---|---|---|---|---|
| | | | [duplex____; ABI____; CTA____; MRA____; DSA____]); Yes____ | |
| Any revascularization for CAD | | | No____ (ischemia-driven____ [symptoms______; stress test______]; based on images____ [CTA____; MRA____; cath____]); Yes____ | |
| Recurrent angina | | | NA | |

Signature: ______________________________ Date: _________________________

# Case Report Form of PIROUETTE-PTA Study (Visit 8)

**Mark**：Yes : ”v” 、No : “ –“ 、Not done: “ND” 、Not applicable : “NA”
**Fill up every item.**

Center ID: _________ Completion: Mandatory Date of form submission:_______

Patient ID: __________

Sex: ____________Age: _____________ Time of Screening: _________________

A. ABI：Lt_____ ；Rt_____ ；# Fontaine class_____ ；Rutherford class_____

B. Symptoms/signs：No_____ ；Yes_____(intermittent claudication_____ ；leg swelling_____；cold foot_____；gangrene______; poor healing wound (ulcer)____; minor tissue loss____; major tissue loss____; others____)

C. Drugs used: Bokey_____ ；Plavix_____ ；Ticagrelor____; Ticocid_____ ；Pletaal_____; Trental_____；Persantin_____; ACEI_____；ARB_____；DRI ; CCB_____( ); Statin_____( ); Fibrate_____( ); TZD_____ ( ); DPP4i_______( ); GLP-1______( ); others (specified )

D.

| | Date of occurrence | Time elapse from Screening (days) | Is it planned staged PCI (or CABG) or PTA (or bypass) | Remarks |
|---|---|---|---|---|
| Symptom-free | | | NA | |
| Intermittent | | | NA | |

| | | | | |
|---|---|---|---|---|
| claudication | | | | |
| Resting pain or numbness | | | NA | |
| Ulcer wound | | | NA | |
| Gangrene | | | NA | |
| Minor amputation | | | NA | |
| Major amputation | | | NA | |
| Total Death | | | NA | |
| CV death | | | NA | |
| Cardiogenic shock | | | NA | |
| Sudden cardiac death | | | NA | |
| Fatal stroke | | | NA | |
| Fatal MI | | | NA | |
| Non-fatal MI | | | NA | |
| Non-fatal stroke | | | NA | |
| CHF hospitalization | | | NA | |
| New ESRD (in pts with no ESRD at baseline) | | | NA | |
| TLR (PAD) | | | No____ (ischemia-driven____ [symptoms____]; | |

| | | | | |
|---|---|---|---|---|
| | | | based on images____ [duplex____; ABI____; CTA____; MRA____; DSA____]); Yes____ | |
| TVR (PAD) | | | No____ (ischemia-driven____ [symptoms____]; based on images____ [duplex____; ABI____; CTA____; MRA____; DSA____]); Yes____ | |
| TLR (CAD) | | | No____ (ischemia-driven____ [symptoms______; stress test______]; based on images____ [CTA____; MRA____; cath____]); Yes____ | |
| TVR (CAD) | | | No____ (ischemia-driven____ [symptoms______; stress test______]; based on images____ [CTA____; MRA____; cath____]); Yes____ | |
| Any Revascularization for PAD | | | No____ (ischemia-driven____ [symptoms____]; based on images____ | |

| | | | | |
|---|---|---|---|---|
| | | | [duplex____;<br>ABI____; CTA____;<br>MRA____;<br>DSA____]); Yes____ | |
| Any revascularization for CAD | | | No____<br>(ischemia-driven____<br>[symptoms______;<br>stress test______];<br>based on images____<br>[CTA____;<br>MRA____;<br>cath____]); Yes____ | |
| Recurrent angina | | | NA | |

Signature: ______________________________ Date: _________________________
